# Improving the blast resistance of the elite rice variety Kongyu-131 by updating the *pi21* locus

**DOI:** 10.1186/s12870-019-1868-x

**Published:** 2019-06-11

**Authors:** Xiaomin Feng, Kangxue Lin, Wenqi Zhang, Jianzong Nan, Xiaohui Zhang, Chen Wang, Rongsheng Wang, Guoqiang Jiang, Qingbo Yuan, Shaoyang Lin

**Affiliations:** 1 State Key Laboratory of Plant Genomics, Institute of Genetics and Developmental Biology, Chinese Academy of Sciences, Beijing, 100101 China; 2 Guangdong Provincial Bioengineering Institute, Guangzhou Sugarcane Industry Research Institute, Guangzhou, 510316 China; 3 University of Chinese Academy of Sciences, Beijing, 100039 China

**Keywords:** Rice, Blast resistance, SNP marker, *Pi21*, Linkage drag

## Abstract

**Background:**

As an elite *japonica* rice variety, Kongyu-131 has been cultivated for over 20 years in the third accumulated temperature zone of Heilongjiang Province, China. However, the cultivated area of Kongyu-131 has decreased each year due to extensive outbreaks of rice blast. To achieve the goals of improving blast resistance and preserving other desirable traits in Kongyu-131, a genome-updating method similar to repairing a bug in a computer program was adopted in this study. A new allele of the broad-spectrum blast resistance gene *pi21* in the upland rice variety GKGH was mined by genetic analysis and introgressed into the genome of Kongyu-131 to upgrade its blast resistance.

**Result:**

QTL analysis was performed with an F_2_ population derived from a cross between Kongyu-131 and GKGH, and a blast resistance QTL was detected near the *pi21* locus. Parental *Pi21* sequence alignment showed that the *pi21* of the donor (GKGH) was a new allele. By 5 InDel or SNP markers designed based on the sequence within and around *pi21*, the introgressed chromosome segment was shortened to less than 634 kb to minimize linkage drag by screening recombinants in the target region. The RRPG was 99.92%, calculated according to 201 SNP markers evenly distributed on 12 chromosomes. Artificial inoculation at the seedling stage showed that the blast resistance of the new Kongyu-131 was improved significantly. Field experiments also indicated that the improved Kongyu-131 had enhanced field resistance to rice blast and grain-quality traits similar to those of the original Kongyu-131.

**Conclusions:**

It is feasible to improve resistance to rice blast and preserve other desirable traits by precisely improving the *Pi21* locus of Kongyu-131. Linkage drag can be eliminated effectively via recombinant selection on both sides of the target gene.

## Background

Rice blast caused by the fungus *Magnaporthe oryzae* is a worldwide rice disease that generally causes a serious reduction in rice yield. The global annual crop loss due to blast was estimated to be $66 billion, representing enough rice to feed 60 million people [1]. Rice blast is a major factor limiting rice yield and affects its grain quality. It is widely accepted that cultivating disease-resistant varieties is the most economical and effective control method.

Discovering and utilizing blast resistance genes are the premises of disease resistance breeding. To date, more than 100 major blast R-genes have been identified. These genes are distributed on all 12 chromosomes of rice, and more than half are concentrated on chromosomes 6, 11 and 12 [2]. Since Wang et al. cloned the first rice blast resistance gene *Pib* [3], more than 30 rice blast resistance genes have been cloned [2]. Except for *bsr-d1* [4], *bsr-k1* [5], *Pid2* [6], *pi21* [7] and *Ptr* [8], the blast resistance genes all encode NBS-LRR proteins. *Pi21* was the first cloned recessive blast resistance QTL, encoding a proline-rich protein that includes a putative heavy metal-binding domain and putative protein-protein interaction motifs [7]. Compared with the susceptible varieties, the variety Owarihatamochi contains 21 bp and 48 bp deletions in the proline-rich motif that lead to an increase in resistance [7]. Cloning of blast resistance genes/QTLs, the discovery of excellent alleles and the elucidation of molecular mechanisms have provided favorable gene resources and a theoretical basis for rice blast resistance breeding.

Conventional breeding for disease resistance combines field phenotype selection and resistance identification to introduce resistance genes by crossing and backcrossing, which is still commonly used in disease resistance breeding. However, the resistance gene is often lost in the process of breeding, which causes low breeding efficiency and a long breeding cycle. With the development of molecular biology and the identification of increasing numbers of blast resistance genes, molecular breeding implementing molecular marker technology has become an important part of plant breeding.

Marker-assisted selection (MAS) can make up for the deficiency of conventional breeding for disease resistance. Combined with conventional breeding methods, MAS has been used to improve resistance to rice blast and bacterial leaf blight [9–11]. However, molecular breeding technology with MAS still has some shortcomings. First, MAS uses DNA markers linked to target genes to indirectly select the target traits. Recombination between the linked markers and target genes can affect the selection accuracy. Second, the introduction of the target gene by MAS can also introduce some unwanted genes, which causes linkage drag. Third, after introducing the target gene by MAS, selection for other traits mainly relies on the field phenotype, which usually causes the loss of some other excellent traits and an unclear genetic background. Solving these problems will help make molecular breeding more accurate and efficient.

Owing to a series of advantages such as early maturity, high quality, high yield, cold tolerance and wide adaptability, Kongyu-131 has been cultivated in over 8 million hectares since its introduction [12]. However, the large-scale planting of Kongyu-131 over decades resulted in a change in the *M. oryzae* population structure and the emergence of dominant virulent strains, which eventually caused an outbreak of blast disease and substantial economic loss. It is unfortunate that Kongyu-131, a precious germplasm resource, had to be withdrawn from production simply because of its susceptibility to rice blast [12]. By resequencing the genome of Kongyu-131 and comparing it with cloned R-gene sequences, Zhang et al. found that Kongyu-131 lacked almost all the R-genes except for *Pish* [13]. By using the principle of genome updating described by Feng et al. [14], we mined a favorable blast resistance gene module (a small chromosome segment hosting a superior *pi21* allelic variant from a blast-resistant upland variety) to improve the blast resistance of Kongyu-131 in this study.

## Results

### Marker-trait association analysis

**Fig. 1:**
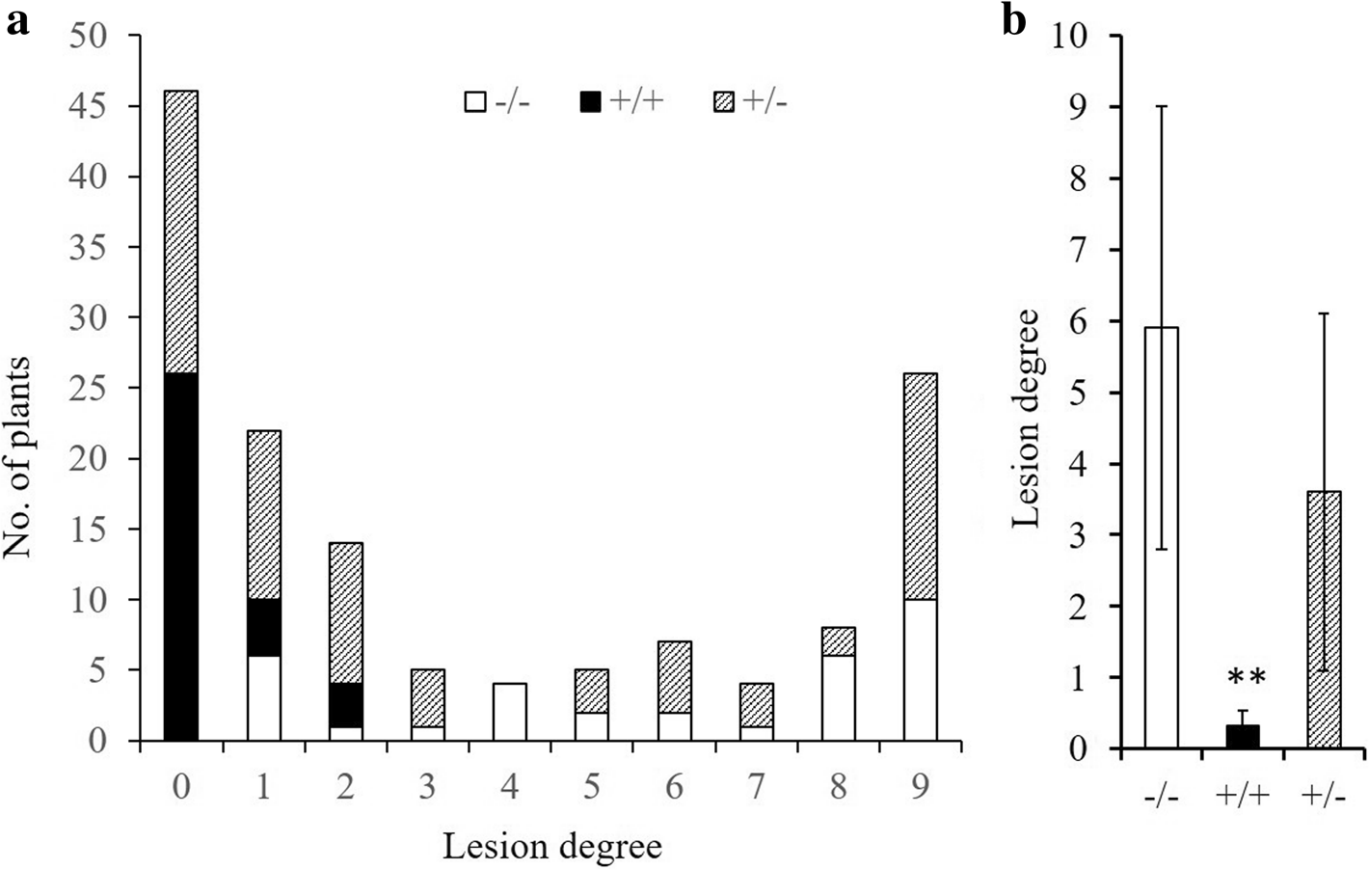
Marker-trait association analysis of the F_2_ population. **a** Frequency distribution of lesion degree in the F_2_ population. **b** Comparison of lesion degree among the three genotypic classes of the *Pi21* locus in the F_2_ population. Data was presented as the means ± SD (standard deviations). −/− represents Kongyu-131-homozygous lines, +/− represents the heterozygote, and +/+ represents GKGH-homozygous lines. **above the bar chart represents a significant difference based on Student’s *t*-test at *p* ≤ 0.01

Genetic analysis was performed by using an F_2_ population derived form Kongyu-131 and GKGH with 141 individuals. The result indicated that the marker tightly linked to *pi21* was significantly associated with LD and explained 16.4% of the phenotypic variation (Table 1). The number of lines with the three genotypic classes at the *pi21* locus (Kongyu-131-homozygous, heterozygous, and GKGH-homozygous genotypes) was 33, 75 and 33, respectively, which showed a good fit to the expected 1:2:1 ratio based on a chi-square test (χ^2^ = 0.57, *p* > 0.75). The frequency distribution for the LD of the three genotypic classes at the *pi21* locus is shown in Fig. 1a. The GKGH-homozygous lines all showed resistance to blast disease, while the LD of both the Kongyu-131-homozygous lines and the heterozygotes ranged from 0 to 9. The average phenotypic value of the GKGH-homozygous lines was significantly lower than that of Kongyu-131, but no difference was observed between the Kongyu-131-homozygous lines and the heterozygote (Fig. 1b).

**Table 1:** QTL for blast resistance in the F_2_ population

Traits	Chr	Linked R-gene	Additive effect	Dominant effect	LOD	Var (%)
LD	4	*pi21*	−2.9	0.5	5.5	16.40%

Additive effect, Dominant effect and LOD were calculated according to the tightly linked SNP marker of *pi21*. Var (%) indicates the percentage of total phenotypic variance explained by the QTL

### Comparison of *pi21* alleles in different rice parents

**Fig. 2:**
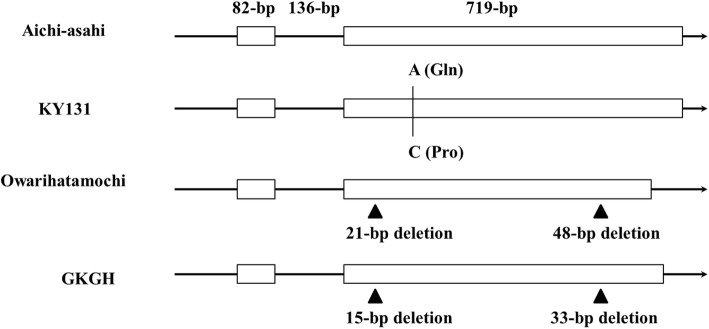
*Pi21* structure and allelic variation of Kongyu-131, GKGH, Aichi-asahi and Owarihatamochi. A SNP in the second exon that resulted in amino acid changes (in parentheses) was identified in Kongyu-131 and is shown as black vertical lines. DNA fragment deletions in the second exon were identified and are shown as black triangles. White rectangles and black horizontal lines represent exons and the untranslated sequence, respectively

The *pi21* sequences of Kongyu-131, GKGH, Aichi-asahi and Owarihatamochi were compared. The resistant *pi21* allele had deletions of 15 bp and 33 bp compared to the susceptible *Pi21* allele in the second exon, corresponding to 21 bp and 48 bp deletions at the same position in a previous report [7]. These results showed that the *pi21* allele in GKGH was a new haplotype. In contrast to the Aichi-asahi allele, the Kongyu-131 allele exhibited a nucleotide change resulting in amino acid variation (Gln to Pro) in the second exon (Fig. 2).

### Foreground selection, recombinant selection, background selection and chromosome composition evaluation

**Fig. 3:**
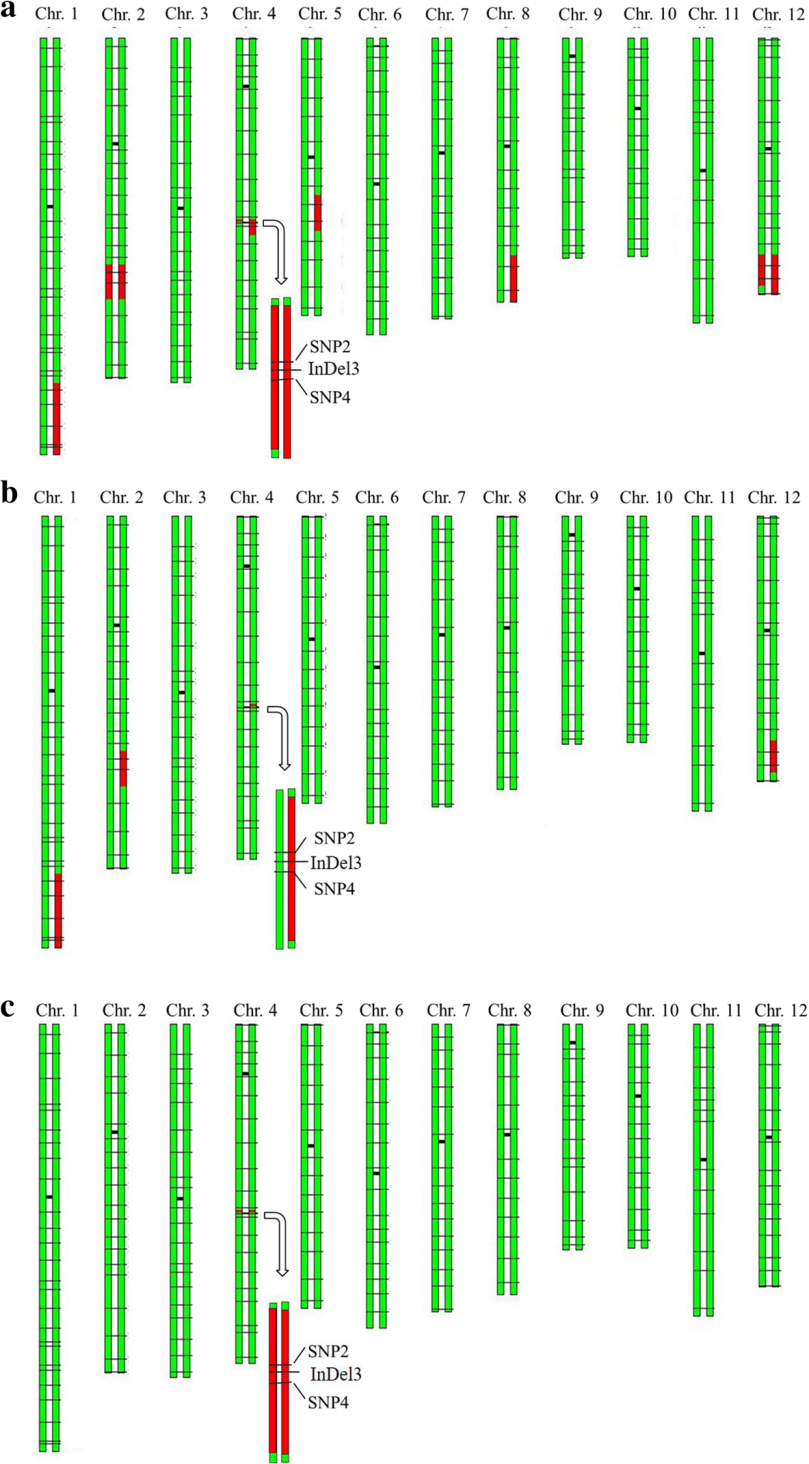
Graphical genotype of the selected lines. **a** BC_3_F_2_-669E10. **b** BC_4_F_1_-30B07. **c** BC_4_F_2_–350E03. The green bars represent the chromosome fragments derived from Kongyu-131, the red bars represent the chromosome fragments derived from GKGH, and the horizontal black lines indicate the SNP markers (the shorter, darker lines represent the centromeres)

Two lines selected from BC_2_F_1_ population were crossed with Kongyu-131 to produce 92 BC_3_F_1_ lines, from which a line named BC_3_F_1_-08G02 in heterozygous form at the InDel3 locus, with recombination on one side (between SNP1 and SNP2) of the target gene, was selected. Selfing was performed to produce 190 BC_3_F_2_ lines, and a line named BC_3_F_2_-669E10, which hosted the target gene and exhibited recombination on the other side (between SNP4 and SNP5), was selected. According to the sequence difference between Kongyu-131 and GKGH, 201 SNP markers evenly distributed on all 12 chromosomes were used to detect the genetic background. The results showed that in addition to the target segment, 5 nontarget segments, including two homozygous segments on chromosomes 2 and 12, were in the background, and the RRPG was 95.59% (Fig. 3a).

To remove the nontarget segments, BC_3_F_2_-669E10 was crossed with Kongyu-131 to produce the BC_4_F_1_ generation with 33 plants, from which a line named BC_4_F_1_-30B07 was selected by using the markers in heterozygous form in BC_3_F_2_-669E10 to genotype the BC_4_F_1_ population. In addition to the target segment, BC_4_F_1_-30B07 hosted 3 nontarget segments on chromosomes 1, 2 and 12, with an RRPG of 98.02% (Fig. 3b). To further remove nontarget segments and obtain the improved line hosting only a very small homozygous segment with a target gene, BC_4_F_2_–350E03 was selected from 948 selfing progenies of BC_4_F_1_-30B07. The length of the target introgression segment in the improved line BC_4_F_2_–350E03 was less than 634 kb, and the RRPG was 99.92% based on 201 SNP markers (Fig. 3c).

### The resistance of the improved line BC_4_F_2_–350E03 to leaf blast at the seedling stage increased significantly

**Fig. 4:**
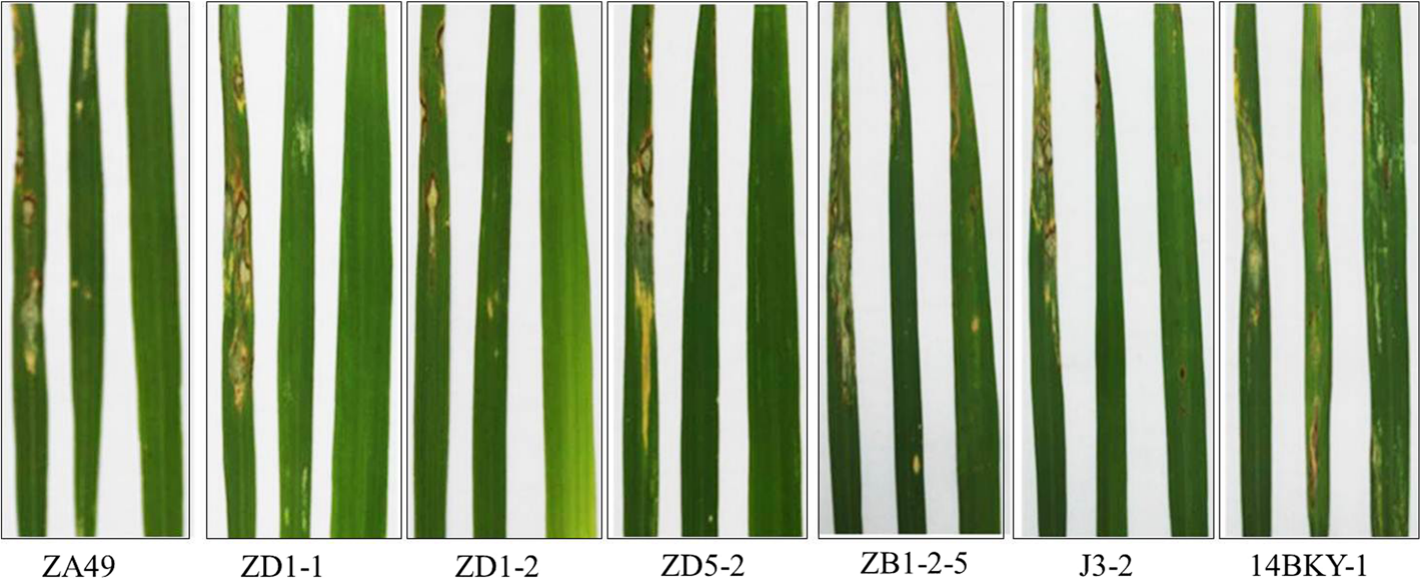
The artificial inoculation results for Kongyu-131, the donor and the improved line BC_4_F_2_–350E03 with the 7 strains of *M. oryzae* at the seedling stage: Kongyu-131 (left), BC_4_F_2_–350E03 (middle), and GKGH (right)

To test the blast resistance effect of the introgressed *pi21* allele from GKGH, two parents and the improved line BC_4_F_2_–350E03 were inoculated with 7 strains isolated from the diseased panicles or leaves of Kongyu-131 at the seedling stage. The results showed that Kongyu-131 was susceptible or highly susceptible to all 7 strains, while the single point substitution line (SPSL) BC_4_F_2_–350E03 showed different degrees of resistance to these 7 strains and a resistance level similar to that of the donor parent (GKGH) (Fig. 4, Table 2). The results indicated that the segment hosting the resistant *pi21* allele from the donor (GKGH) significantly improved the blast resistance of Kongyu-131 to the 7 strains from Heilongjiang Province.

**Table 2:** The artificial inoculation results for Kongyu-131, the donor and the SPSL BC_4_F_2_–350E03 with the 7 isolates of *M. grisea* at the seedling stage

Isolate		Lesion degree	
	KY131	BC_4_F_2_–350E03	GKGH
ZA49	7.0 ± 0.8	3.0 ± 0.7	0.4 ± 0.4
ZD1–1	8.5 ± 0.7	2.4 ± 0.8	0.2 ± 0.4
ZD1–2	7.1 ± 1.0	2.2 ± 0.9	0.3 ± 0.5
ZD5–2	7.9 ± 0.9	2.0 ± 0.8	0.3 ± 0.5
ZB1–2-5	8.6 ± 0.7	2.9 ± 0.9	2.5 ± 1.0
J3–2	8.4 ± 0.7	2.1 ± 0.9	1.2 ± 0.6
14BKY-1	8.4 ± 0.8	2.9 ± 0.9	2.4 ± 1.0

Data presented as the means ± SD (standard deviations) were obtained from 20 plants with two replicates

### No significant difference in agronomic traits was observed between Kongyu-131 and the improved line BC_4_F_2_–350E03

**Fig. 5:**
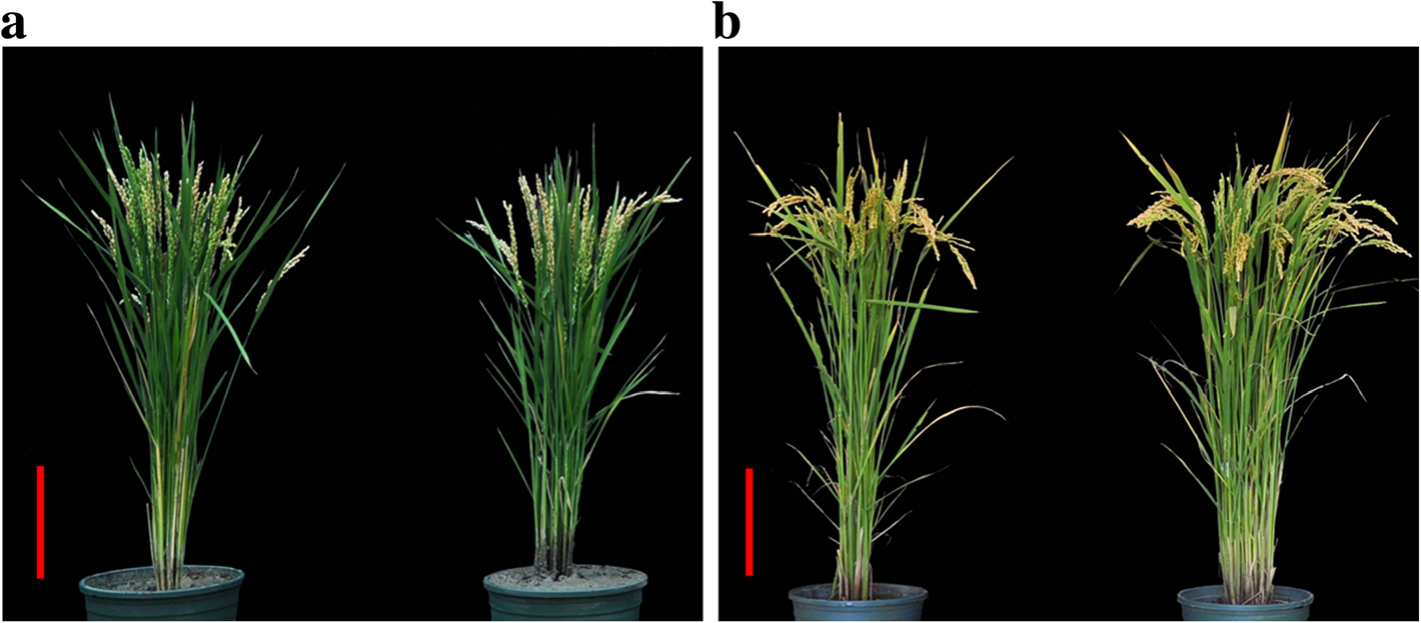
Plant architecture of Kongyu-131 and the improved line BC_4_F_2_–350E03. **a** in Beijing in 2017 and **b** in Jiamusi in 2017; Kongyu-131 (left) and BC_4_F_2_–350E03 (right). The scale bar represents 20 cm

The plant architectures of Kongyu-131 and the improved line BC_4_F_2_–350E03 in Beijing and Jiamusi are shown in Fig. 5, and the comparison of agronomic traits is shown in Table 3. In Beijing, the DTH of the SPSL was 2.7 days later than that of Kongyu-131, while other traits such as PH, YP, SSP and TGW of the improved line were similar to those of Kongyu-131. In Jiamusi in 2017 and 2018, no significant difference was observed for important agronomic traits such as DTH, PH, SSP, YP and TGW between the improved line and Kongyu-131. However, the GYP of the improved line was significantly higher than that of Kongyu-131 because some plants of Kongyu-131 were infected with rice blast, while the improved line was free from blast disease. These results indicated that the introgression of the resistant *pi21* allele from the donor parent significantly improved the field blast resistance of Kongyu-131.

**Table 3:** Agronomic performance of Kongyu-131 and the improved line BC_4_F_2_–350 E03 in three environments

Trait	2017 Beijing		2017 Jiamusi		2018 Jiamusi	
	KY131	BC_4_F_2_–350E03	KY131	BC_4_F_2_–350E03	KY131	BC_4_F_2_–350E03
DTH	66.2 ± 0.7	68.9 ± 1.4^**^	103.3 ± 1.4	103.6 ± 1.5	102.8 ± 1.4	103.1 ± 1.5
PH (cm)	50.2 ± 0.8	49.7 ± 1.1	74.0 ± 3.5	74.7 ± 3.1	74.3 ± 4.7	74.2 ± 3.1
ETP	29.1 ± 1.5	28.4 ± 2.4	34.7 ± 3.5	33.5 ± 4.0	30.7 ± 4.5	32.2 ± 4.9
PL (cm)	13.3 ± 0.5	13.0 ± 0.8	16.1 ± 0.6	16.0 ± 0.7	16.0 ± 1.7	16.3 ± 1.2
NPB	6.7 ± 0.8	6.8 ± 0.6	10.2 ± 0.9	10.6 ± 1.2	11.7 ± 1.5	11.2 ± 1.0
GNP	67.4 ± 6.2	70.6 ± 6.8	116.3 ± 8.1	120.3 ± 14.1	102.9 ± 4.7	101.3 ± 6.3
SSP (%)	94.7 ± 2.7	93.1 ± 2.5	94.6 ± 3.2	93.6 ± 2.0	95.6 ± 1.2	95.8 ± 1.1
YP (g)	29.3 ± 2.7	28.7 ± 2.4	53.0 ± 2.3	54.2 ± 2.8	49.4 ± 2.3	52.4 ± 3.5
TGW (g)	23.9 ± 0.3	23.9 ± 0.8	27.2 ± 0.6	27.3 ± 1.0	27.0 ± 1.0	27.0 ± 1.0
GYP (kg)	NA	NA	NA	NA	3.97 ± 0.26	4.49 ± 0.34^**^

Data presented as the means ± SD (standard deviations) were obtained from 10 plants with three replicates under natural conditions in Beijing and Jiamusi. The planting density was 30 cm × 20 cm, with one plant per hill. GYP data were obtained from plants in 10 plots, and the area per plot was 5.76 m^2^ (2.4 m × 2.4 m). ^**^represents a significant difference at *p* ≤ 0.01 based on Student’s *t*-test. “NA” means data not available

### The quality traits of the improved line were similar to those of Kongyu-131

**Fig. 6:**
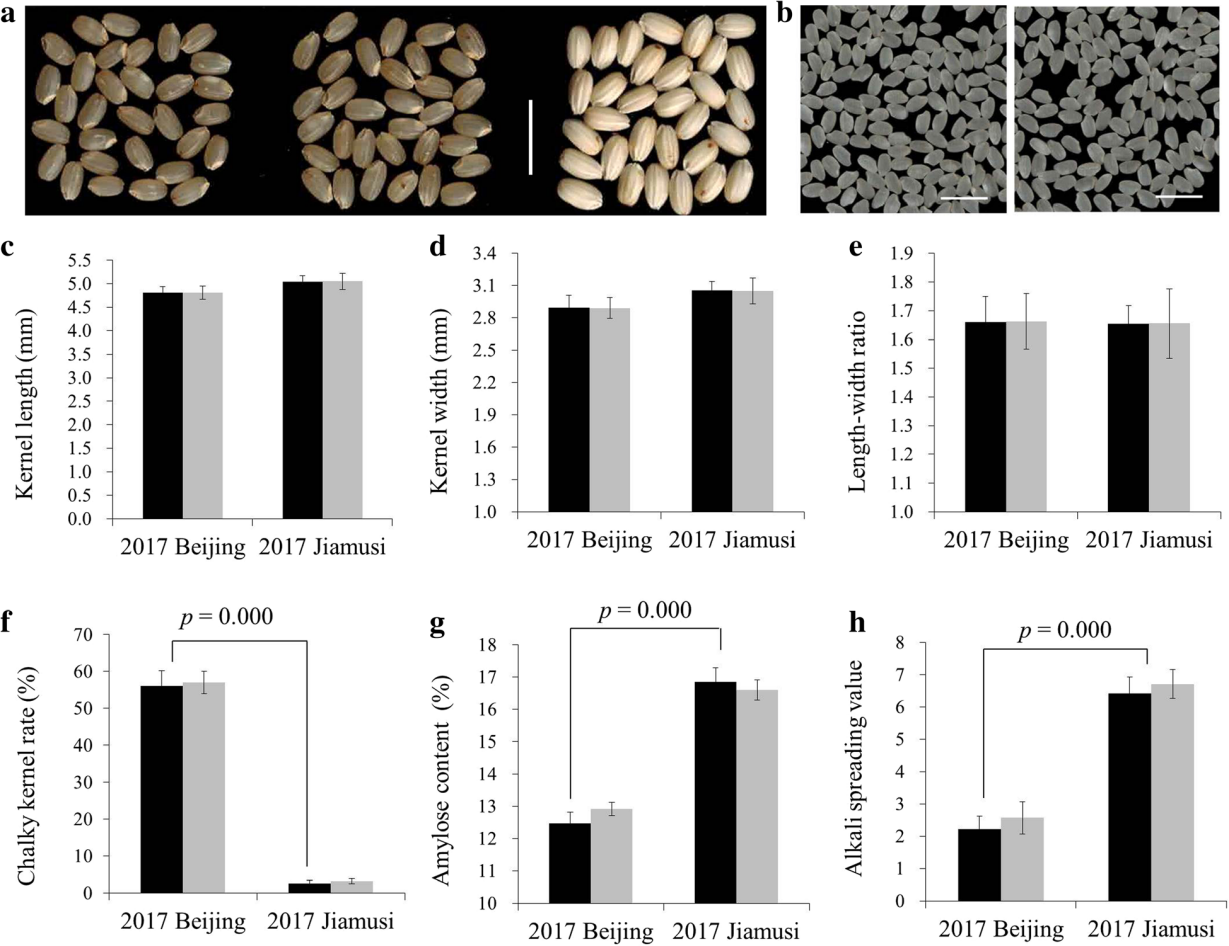
Comparison of grain quality in the improved line and Kongyu-131. **a** Performance of the brown rice cultivar. From left to right, Kongyu-131 in Jiamusi in 2017, the SPSL BC_4_F_2_–350E03 in Jiamusi in 2017, and the donor GKGH in Beijing in 2017 (normal heading did not occur in Jiamusi in 2017). **b** Performance of the milled grain. Kongyu-131 (left), BC_4_F_2_–350E03 (right); the scale bar represents 1 cm. **c** Kernel length (*n* = 30). **d** Kernel width (*n* = 30). **e** Length-width ratio (*n* = 30). **f** Chalky kernel rate (*n* = 500). **g** Amylose content (*n* = 4). **h** Alkali spreading value (*n* = 14). In **c**-**h**, the black bar represents Kongyu-131, and the gray bar represents the improved line BC_4_F_2_–350E03. Data was presented as the means ± SD (standard deviations)

To determine whether the improved line retained the original high-quality characters, we first compared the appearance quality traits, namely, KL, KW, LWR and CKR, to those of Kongyu-131 (Fig. 6). The improved line exhibited appearance quality traits similar to those of Kongyu-131 in the same location (Fig. 6a-f). However, the CKRs of the improved line and Kongyu-131 in Beijing were both significantly higher than in Jiamusi (Fig. 6f). Then, we compared the cooking quality, namely, the AC and ASV, of the improved line to that of Kongyu-131 (Fig. 6g, h). No significant differences in AC and ASV were detected between the lines at the same location. However, the AC and ASV of both the improved line and Kongyu-131 in Beijing were significantly lower than those in Jiamusi.

## Discussion

The large rice blast outbreak in the paddy fields where the elite cultivar Kongyu-131 is cultivated has led to the gradual withdrawal of this cultivar from production. It is challenging to use a blast-resistant upland rice variety as the donor parent to improve the blast resistance of Kongyu-131 and avoid changing other desirable characteristics. Therefore, we tried a precise and targeted genome-updating method [15, 16], namely, only updating the susceptible *Pi21* locus in the Kongyu-131 genome, to improve the blast resistance of this cultivar. Ultimately, the updated Kongyu-131 hosting the resistance allele of *pi21* from the donor parent GKGH exhibited high blast resistance. Moreover, the results of the field experiment showed that the agronomic characteristics and quality traits of the updated Kongyu-131 in Beijing and Jiamusi were similar to those of the original Kongyu-131.

The genetic analysis results in F_2_ population were consistent with *pi21* being a recessive rice blast resistance gene. But the total number of heterozygotes shown resistance to blast disease (with LD of 0–3) even exceeded that of GKGH-homozygous lines (Fig. 1). This could be explained by the existence of other unidentified blast resistance genes in the genetic background or it is possible that some plants may be just escaped during the disease test. CKRs of the improved line and Kongyu-131 in Beijing were both significantly higher than in Jiamusi (Fig. 6f), this result may be explained by the fact that the latitude of the Beijing area was not in the suitable zone for the cultivation of Kongyu-131, and the high accumulated temperature caused early flowering and rapid, insufficient grain filling of Kongyu-131.

### From field selection by phenotype to indoor selection by genotype

Traditional breeding of rice disease resistance mainly relied on phenotype selection according to comprehensive field characteristics, including resistance performance, which usually caused the loss of resistance genes during the breeding process. In addition, identification results were easily affected by the environment, and field selection was constrained by the individual developmental stage, which led to low selection accuracy and efficiency. Instead of traditional breeding, in this study, after determining that the donor parent GKGH contained the superior *pi21* allele, foreground selection, recombinant selection and background selection were performed to select suitable plants using 201 SNP markers, including SNP1-SNP5, at the seedling stage. Selection based on genotype was used as a substitute for field phenotype selection, which made the selection results reliable and independent of environmental impact.

### Hastening the RRPG and removing linkage drag

Targeted improvement of an undesirable trait has always been the goal of breeders. However, due to technical limitations, traditional breeding methods often lose some other desirable traits while improving target traits. An unknown genetic background makes the goal of only improving the undesirable trait hard to realize. In this study, the background was detected by 201 SNP markers evenly distributed throughout the genome to screen out the individuals with the target gene and highest RRPG. Then, the selected lines were crossed with Kongyu-131 to accelerate the RRPG. In addition to backcrossing, this study also used the principle of selfing separation to speed up the RRPG and shorten breeding time. After 5 crosses and two self-crosses combined with selection by genotype, the improved line BC_4_F_2_–350E03 was finally selected with the resistant *pi21* allele from the donor parent GKGH, and the RRPG reached 99.92%.

The target chromosome segment was shortened to less than 634 kb via two rounds of recombinant selection on both sides of the *pi21* locus. The actual size of the introduced segment should be further confirmed by adding markers between SNP1 and SNP2 and between SNP4 and SNP5. According to a previous report, a closely linked gene downstream of *pi21*, Os04g0401400 (LOC_Os04g32890), was associated with inferior eating quality, which hampered the application of the *pi21* blast resistance gene in breeding [7]. In this study, the resistant *pi21* allele from the donor GKGH was used to replace the corresponding allele in the Kongyu-131 genome, and two rounds of recombinant selection were performed to minimize linkage drag near the target gene. Finally, the quality traits of the updated Kongyu-131 were found to be similar to those of Kongyu-131, which indicated that the linkage drag was effectively removed.

### Seedling blast resistance and adult blast resistance

Rice blast may occur at different stages of rice growth and in different parts of rice. Leaf blast and panicle blast are the most common and most harmful types of blast. However, there is no agreement among reports on whether there is consistency between seedling blast resistance and adult blast resistance [7, 17]. The difference may be attributed to differences in the introduced target R-gene and genetic background. Moreover, leaf and panicle tissues are observed at different developmental stages, and the expression of R-gene has spatiotemporal specificity [18]. *Pb1* transcript levels increased during the development of *Pb1+* cultivars; this expression pattern accounted for their panicle resistance [19]. Inoue et al. found that *Pb1* was negatively dependent on at least three QTLs on chromosomes 7, 9 and 11 and positively dependent on one QTL on chromosome 8 in the genome of the rice cultivar Kanto209 [20]. In this study, artificial inoculation at the seedling stage was carried out to identify the rice blast resistance level of the improved line and two parents. The leaf blast resistance level of the improved line was enhanced significantly. The field investigation in Jiamusi, Heilongjiang Province, showed that some Kongyu-131 plants were infected with panicle blast, while the improved line was immune to blast disease. Further validation of the panicle blast resistance under nursery conditions is needed.

### Strategies for the loss of resistance to rice blast

To date, most of the cloned R-genes mediate specific resistance. However, blast-resistant cultivars easily lose resistance due to frequent changes in *M. grisea* pathogenicity [21, 22]. Therefore, identifying durable disease resistance genes such as *pi21* and *Pb1* for disease resistance breeding has been a goal of breeders for several decades.

In addition to durable blast resistance genes, the complementarity of multiple R-genes can be utilized comprehensively to effectively avoid the defects that accompany single gene-mediated resistance. At present, the introduction of multiple resistance genes into a single variety is widely used by breeders to improve disease resistance [10, 17, 23–25].

The concept of multiline was well demonstrated in the management of wheat rust diseases [26], and more recently, a transgenic multiline variety for controlling powdery mildew of wheat has been developed [27]. A previous study found that Kongyu-131 contained the resistant *Pish* gene but lacked almost all other cloned blast resistance genes [13]. The method of improving the *Pi21* locus in Kongyu-131 described here could be used to update other blast resistance genes. Then, SPSLs carrying individual blast resistance genes in the genetic background of Kongyu-131 can be developed with nearly identical height, maturity, plant architecture, grain and cooking quality traits and other characteristics. These SPSLs are similar in appearance and genetic constitution but carry different genes for resistance to blast disease. Therefore, the lines can be used to create a multiline variety by mechanically mixing their seeds. This method also offers farmers a way to treat outbreaks of blast disease. Once a line’s resistance is overcome in the field, it is easy to analyze its R-genes and replace them with other blast resistance genes.

The large-scale cultivation of an excellent rice variety over the long term tends to cause the loss of blast resistance. Therefore, in addition to the application of R-genes, it is also necessary to monitor the pathogenic races and virulence gene structure of the *M. oryzae* population in the rice cultivation area to update rice varieties with corresponding genes for blast resistance.

## Conclusions

The cultivation of blast-resistant rice varieties is the most economical and effective method to control rice blast. In this study, an upland rice variety (GKGH) with broad-spectrum resistance to blast disease was used as the donor parent to improve the resistance level of Kongyu-131. The resistant *pi21* allele was used to precisely substitute the corresponding allele into Kongyu-131. Five SNP markers in and around *pi21* were designed to break the linkage drag by two rounds of recombinant selection on both sides of the target gene. Finally, the newly bred Kongyu-131 exhibited improved blast resistance both in an incubator under artificial inoculation and in natural conditions with no yield or quality penalties. The methods of updating varieties and eliminating linkage drag proposed in this study can also provide a reference for the improvement of other crop varieties.

## Methods

### Rice materials

The recurrent parent Kongyu-131 (originated in Japan and introduced into China as an elite rice variety; the seeds were from Lin’s lab in the Institute of Genetics and Developmental Biology, Chinese Academy of Sciences) is an early-maturity *japonica* rice variety grown in the high-latitude zone of Heilongjiang Province, China. This variety has strong tillering ability, lodging resistance, and cold tolerance but the defect of high blast susceptibility. Kongyu-131 requires an accumulated temperature of 2330 °C and produces an average yield of 7684.5 kg/ha. The donor parent GKGH (originated in China, and the seeds were from Lin’s lab in the Institute of Genetics and Developmental Biology, Chinese Academy of Sciences) is an upland rice variety resistant to rice blast, but normal heading does not occur in this variety in Heilongjiang Province due to an unsuitable photoperiod and temperature.

### Isolates

Diseased panicles and leaves of Kongyu-131 were collected from paddy fields of rice production areas in Heilongjiang Province from 2002 to 2006 and used to isolate blast fungus [12]. Five genetically different and representative isolates (ZA49, ZD1–1, ZD1–2, ZD5, and ZB1) were used in this study. In addition, two other strains (J3–2 and 14BKY-1) were isolated from the diseased panicles or leaves of Kongyu-131 from paddy fields in 2013 and 2014, respectively. All 7 isolates were used for the present study.

### Resequencing, comparison of *Pi21* sequences and SNP marker design

The Kongyu-131 and GKBR rice varieties were sequenced using a HiSeq 2000 sequencing system (Illumina, USA). We downloaded the *Pi21* DNA sequences of Aichi-asahi and Owarihatamochi [7] from GenBank (http://www.ncbi.nlm.nih.gov/genbank) and compared the *Pi21* DNA sequences of Kongyu-131, GKGH, Aichi-asahi and Owarihatamochi using DNAMAN software. SNP markers were developed according to the SNPs between Kongyu-131 and GKGH. The SNPs and their flanking 22~24 nt sequences were used to design locus-specific or allele-specific primers, and the amplicons were approximately 50~100 bp long. Five InDel/SNP markers named SNP1-SNP5 were designed (Table 4). InDel3 was an InDel marker located in *Pi21*, SNP1 and SNP2 were located upstream of *Pi21*, and SNP4 and SNP5 were located downstream of *Pi21*. SNP2 was close to the 5′-untranslated region (UTR) of *Pi21,* and SNP4 was close to the 3′-UTR of *Pi21*. The distance between SNP2 and SNP4 was 4692 bp, while the distance between SNP1 and SNP5 was 634 kb.

**Table 4:** Sequences and positions of the SNP markers developed for the selection of *pi21*

Markers	Chr.	Position (IRGSP1.0)	Forward primer (5′-3′)	Reverse primer (5′-3′)
SNP1	4	19,354,482	TGCACCAGTATTAACAATTGA	CCATAAAACTAGATATAGAGC
SNP2	4	19,855,434	TGCTAATCCGGAATCTCGAC	TTCGAACATAAGGTGGTCGAC
InDel3	4	19,856,847	GATCCTCATCGTCGACGTCT	TTGCAGTCCTCCGGAGGCTTCT
SNP4	4	19,860,126	TGGGATAGTCATCAATGGTGC	CCATTGATGAGTCTATTGTAG
SNP5	4	19,988,873	ATAATGGGATGAGACCCATC	TGGGATCCAGATTCGTAGTCTC

### Population development and line selection by genotyping

**Fig. 7:**
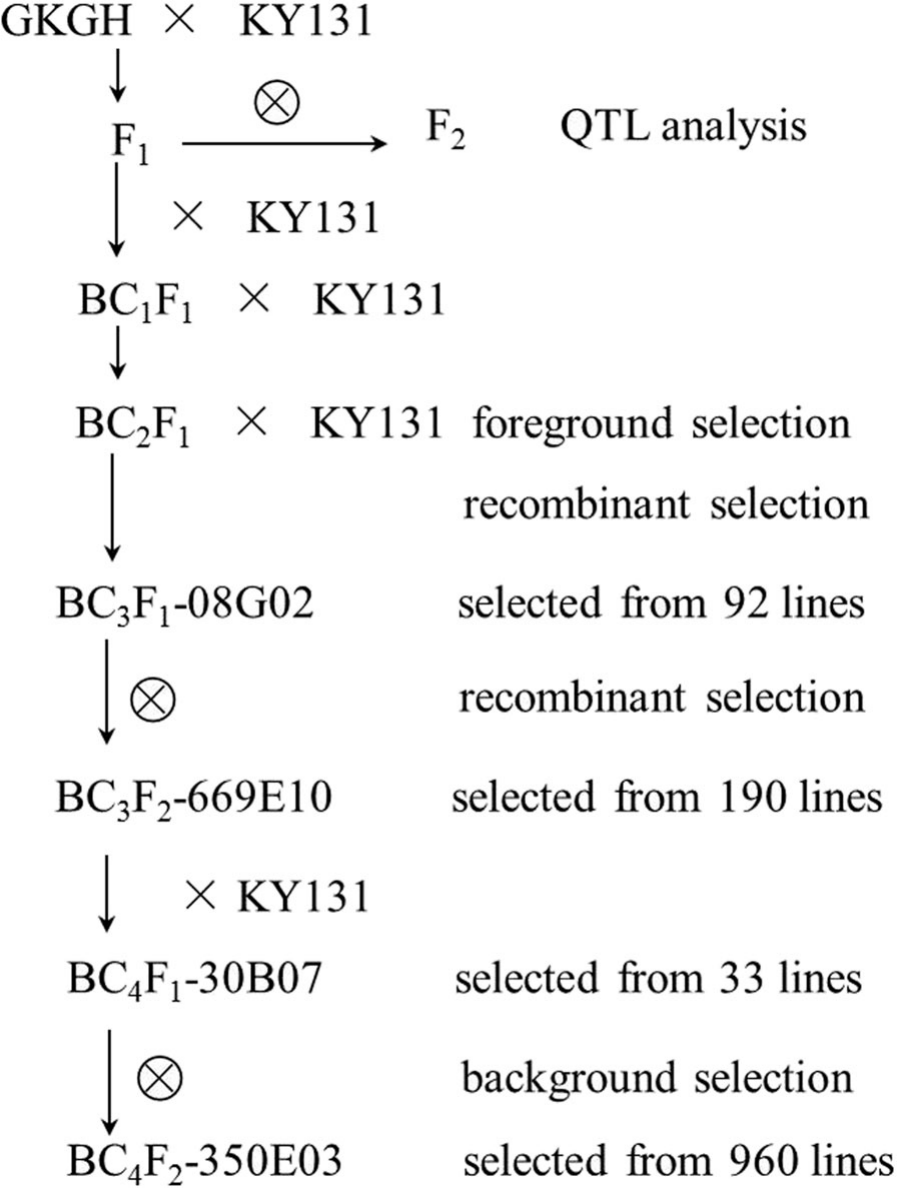
Procedure for population development

The donor parent GKGH was crossed and backcrossed with the recurrent parent Kongyu-131 to produce a series of populations in Hainan and Guangdong Provinces from 2013 to 2016 (Fig. 7). The F_2_ population was used for QTL analysis. Two lines hosting the target gene were selected from the BC_2_F_1_ population according to the genotype at InDel3. The line named BC_3_F_1_-08G02, with crossover on one side of the target gene, was selected from the backcross progenies of the two lines. The line named BC_3_F_2_-669E10, with crossover on the other side of the target gene, was selected from the selfed progenies. To further eliminate the nontarget chromosomal fragments from the background, BC_3_F_2_-669E10 was crossed with Kongyu-131, and a line named BC_4_F_1_-30B07, which hosted the target gene and the minimum number of nontarget segments, was selected from the progenies. Ultimately, a line named BC_4_F_2_–350E03, with only one small chromosome fragment carrying the target gene, was selected from a large BC_4_F_2_ population derived from BC_4_F_1_-30B07.

### Inoculation and phenotyping

The F_2_ population (141 lines) derived from Kongyu-131 and GKGH was seeded into a specially made 96-well PCR plate with a hole (Φ = 2.5 mm) at the bottom. One seed was placed in each well with the embryo pointing upward, and seedlings at the 3~4 leaf stage were placed in a climatic chamber for inoculation. Inoculations were performed by spraying conidial suspensions (5 × 10^5^ spores/ml) onto the leaves until run-off occurred. Before inoculation, 0.05% Tween 20 was added to the suspension to increase the adhesion of the spores to the plant. The relative humidity was maintained at 100% for 24 h of dark treatment at 25–28 °C before transferring the plants back to the greenhouse. After 7 days, the lesion degree (LD) of the rice leaves was scored from 0 to 9 according to the Standard Evaluation System of the International Rice Research Institute (IRRI) [28]. The lines with scores of 0–3 were considered to be resistant to blast disease, while those with scores of 7–9 were considered to be susceptible to blast disease. The methods for evaluating the resistance of the improved line BC_4_F_2_–350E03 and two parents were the same as those described above, with two duplications. Each duplication included 7 specially made PCR plates, with Kongyu-131 in rows A and E, the donor parent GKGH in rows D and H, and BC_4_F_2_–350E03 in the other 4 rows. Seven isolates (ZA49, ZD1–1, ZD1–2, ZD5, ZB1, J3–2 and 14BKY-1) were used for inoculation.

### Genotyping and data analysis

DNA extraction, PCR and high-resolution melting (HRM) analysis were carried out as described by Feng et al. [14]. According to the physical position of the cloned blast resistance genes and the SNPs between Kongyu-131 and GKGH, 12 SNP markers tightly linked to corresponding blast resistance genes were developed to genotype the F_2_ population. A chi-square test was used to analyze the F_2_ population segregation data. Marker-trait association analysis was performed using Mapmaker/QTL 1.1b [29] with a threshold logarithm of the odds (LOD) score of 2.0.

### Evaluation of agronomic traits

The improved line BC_4_F_2_–350E03 and Kongyu-131 were planted in Jiamusi (E130°57′, N46°23′), Heilongjiang Province, and in Beijing (E116°30′, N39°95′) with three replicates. Each replicate consisted of 8 rows with 12 plants per row. The plant spacing was 20 cm within each row and 30 cm between adjacent rows, and the field management was the same as that used in local paddy fields. At the time of harvest, 10 plants in the middle of each plot were selected randomly for phenotypic investigation; every selected plant had to meet the condition that its surrounding 8 plants exhibited normal growth vigor. Days to heading (DTH) was calculated from the time of seed soaking to the time when 50% of plants flowered in the plots. Plant height (PH), effective tillers per plant (ETP), panicle length (PL), number of primary branches per panicle (NPB), grain number per panicle (GNP), thousand-grain weight (TGW), yield per plant (YP), grain yield per plot (GYP) and moisture content of the grain were investigated. The YP and GYP at a moisture content of 15% and the seed setting percentage (SSP) were calculated.

### Evaluation of quality traits

To assay the grain characteristics, ten fully filled grains from each plot were randomly selected to measure kernel length (KL) and kernel width (KW) using a Vernier caliper. The length-to-width ratio (LWR) was calculated as grain length divided by grain breadth. One hundred fully filled grains were randomly selected with 5 replicates to determine the chalky kernel rate (CKR). The alkali spreading value (ASV) was tested by soaking seven whole-milled grains of the improved line and Kongyu-131 in 10 ml of 1.7% KOH arranged with equal spacing in Petri plates. The Petri plates were kept at 30 °C for 24 h; thereafter, the grains were individually scored on a scale of 1–7 as described by Khanna et al. [11]. The amylose content (AC) was measured as described by Tian et al. [30] with four replicates and two parallel measurements.

Experimental research on plants in this study complied with institutional, national, or international guidelines. Field studies were conducted in accordance with local legislation. All the rice populations in this study were developed by technicians in our laboratory with crossing or selfing, and the materials were non-transgenic plants, no permissions and/or licenses were required.

## Data Availability

The dataset supporting the conclusions of this article is included within this article.

## Abbreviations

AC: Amylose content
ASV: Alkali spreading value
CKR: Chalky kernel rate
DTH: Days to heading
ETP: Effective tillers per plant
GNP: Grain number per panicle
GYP: Grain yield per plot
HRM: High-resolution melting
KL: Kernel length
KW: Kernel width
LD: Lesion degree
LWR: Length-to-width ratio
MAS: Marker-assisted selection
NPB: Number of primary branches per panicle
PH: Plant height
PL: Panicle length
QTL: Quantitative trait locus
RRPG: Recovery ratio of recurrent parent genome
SNP: Single nucleotide polymorphism
SPSL: Single point substitution line
SSP: Seed setting percentage
TGW: Thousand-grain weight
UTR: Untranslated region
YP: Yield per plant
